# Reconstruction using low-porosity β-tricalcium phosphate for a chondroblastoma in the proximal humeral epiphysis

**DOI:** 10.1093/jscr/rjac526

**Published:** 2022-12-05

**Authors:** Akio Sakamoto, Takashi Noguchi, Shuichi Matsuda

**Affiliations:** Department of Orthopaedic Surgery, Graduate School of Medicine, Kyoto University, Kyoto, Japan; Department of Orthopaedic Surgery, Graduate School of Medicine, Kyoto University, Kyoto, Japan; Department of Orthopaedic Surgery, Graduate School of Medicine, Kyoto University, Kyoto, Japan

## Abstract

Chondroblastoma is a locally aggressive tumor, commonly occurring in the epiphysis of long bones. Damage after curettage at the joint surface can occur. Low porosity β-tricalcium phosphate (β-TCP) blocks are characterized by their high compression resistance. Reconstruction in which low-porosity β-TCP blocks are used as a strut have been reported for bone tumors around the knee. In the current report, a 13-year-old female with a chondroblastoma that extended to the subchondral bone of the proximal humeral epiphysis was treated with curettage and strut reconstruction using low-porosity β-TCP blocks. The implanted β-TCP blocks were incorporated without damage or shoulder dysfunction. Application of the strut reconstruction method using low-porosity β-TCP blocks is suitable for a chondroblastoma of the epiphysis in the humerus.

## INTRODUCTION

Chondroblastoma is a locally aggressive tumor occurring primarily in adolescents and young adults. Chondroblastomas are typically found in the epiphyses and apophyses of long bones and extend to the subchondral bone. The most common sites at which chondroblastomas localize are at the proximal tibia, distal femur, proximal femur and the proximal humerus [1, 2]. Chondroblastomas appear as an osteolytic lesion in radiographs. Clinical manifestations include pain, local swelling, effusion and limited motion at the affected joint [1, 2].

In most cases, chondroblastomas are treated by curettage. The filler after curettage is typically methyl methacrylate (bone cement), tricalcium phosphate (TCP) and either an autologous or allogenic bone graft [3–5]. Because bone cement lacks remodeling ability, subsequent bone incorporation cannot be expected. Furthermore, treatment with bone cement as a filler is associated with the risk of degenerative changes in the area of the subchondral lesion, possibly due to the *‘*heat’ of polymerization [6]. Conversely, TCP will lack mechanical strength prior to bone consolidation.

Low-porosity β-TCP demonstrates strong compressive resistance. A reconstruction method that utilizes low-porosity β-TCP blocks has been reported for giant cell tumors of bone (GCTB) in the knee [7]. In the current case study, reconstruction using low-porosity β-TCP was the treatment for a humeral chondroblastoma.

## CASE REPORT

**Figure 1:**
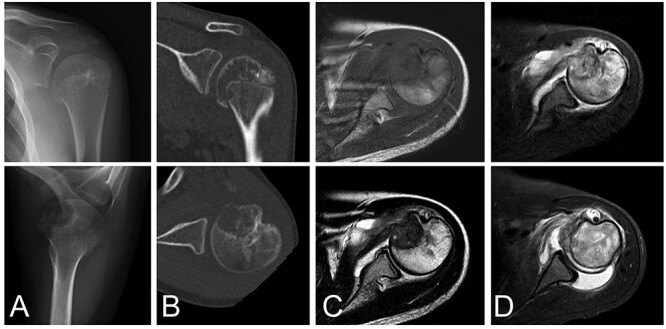
Chondroblastoma in left humerus of a 13-year-old female. Plain radiographs **(A)** and commuted tomography (CT) scans **(B)** show osteolysis with marginal sclerosis in the humeral epiphysis. Calcification is visible in CT images (B). The lesion has homogenous low signal intensity on the T1-weighted image (C-top) and heterogeneous signal intensity on the T2-weighted image (**C**-bottom) using magnetic resonance images.

A 13-year-old female who reported left shoulder pain for a year was referred to our institute after she visited a hospital nearby. Her range of shoulder motion was restricted due to pain and tenderness observed over the anterior shoulder. Plain radiographs showed an osteolytic lesion at the anterior humeral epiphysis extending into the subchondral bone (Fig. 1A). Computed tomography (CT) was used to detect an osteolytic lesion with clear margins, and the absence of subchondral bone was indicative of tumoral extension. Calcification within the lesion was observed on the CT image (Fig. 1B). Magnetic resonance imaging (MRI) of the lesion revealed homogenous low signal intensity on T1-weighted images and heterogeneous low signal intensity with partial high foci on T2-weighted images. Bone marrow edema with high signal intensity on T2-weighted images was observed around the lesion. Fluid collection was also evident at the shoulder joint (Fig. 1C–D). Under general anesthesia, an open biopsy was performed, and the diagnosis of chondroblastoma was confirmed. Histological features included a proliferation of round or polygonal cells with well-defined cytoplasm, an eccentric nucleus, and scattered osteoclast-like giant cells.

**Figure 2:**
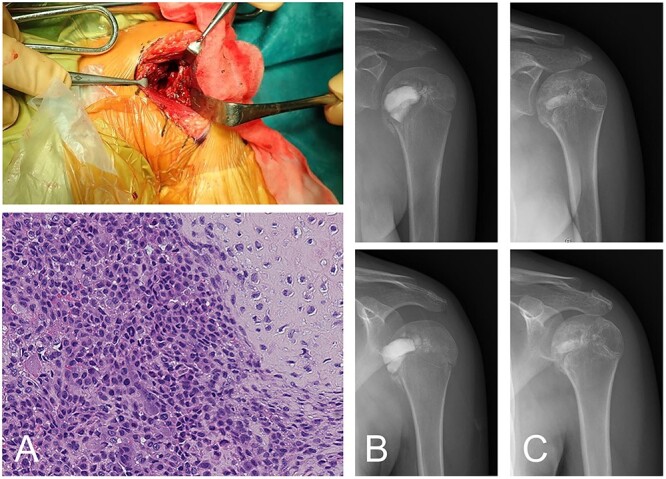
Chondroblastoma in left humerus of a 13-year-old female (same case as Fig. 1). The lesion was curetted via a deltopectoral approach (**A**-top). Histology shows cartilaginous differentiation with the proliferation of round or polygonal cells and a well-defined cytoplasm and eccentric nucleus (A-bottom). Osteoclast-like giant cells were also observed. The cavity after curettage was filled with low-porosity β-TCP blocks and normal porosity particles (**B**). The implanted β-TCP was stable and by 2 years 5 months after surgery was almost entirely incorporated into the surrounding bone (**C**).

Following the diagnosis of chondroblastoma, curettage was performed under general anesthesia. Via a deltopectoral approach, the subscapularis muscle was cut at the shoulder joint and the anterior aspect of the humeral head was revealed. The anterior cortex had softened. Subchondral bone at the lesion was not observed at the joint side of the lesion. After curettage, potential residual tumors along the cavity wall in the host’s bone were burned using an electrical knife; however, the joint side remained untreated in order to avoid damaging articular cartilage. An ethanol adjuvant was not used because the patient could have had an allergic reaction to the alcohol. For the cavity, standard β-TCP particles were positioned in the subchondral space, then low-porosity β-TCP blocks were implanted as a strut and standard β-TCP particles were added to fill the space. The subscapularis muscle was sutured and reconstructed. The diagnosis of chondroblastoma was confirmed following a histological examination of the tumor tissue (Fig. 2A and B).

Rehabilitation to regain range of motion began the day after surgery. Rehabilitation progress was made in accordance with controllable pain. Full range of motion in the shoulder joint was accomplished without any complications 6 months after surgery. The implanted β-TCP used to treat chondroblastoma was incorporated gradually, and no recurrence was observed 2 years and 5 months after surgery, by which time the replacement of β-TCP was almost complete (Fig. 2C).

## DISCUSSION

The treatment of chondroblastoma can involve intralesional curettage and defect reconstruction with an autologous bone graft [8, 9]. Reported local recurrence rates after curettage vary from ~10% to >30% [1, 10]. Adjuvants for aggressive chondroblastomas include phenol, ethanol or hydrogen peroxide [2, 4]. Aggressive curettage, such as with a high-speed burr, is performed to reduce possible recurrence; although it could damage the joint cartilage and the growth plate because the majority of chondroblastomas are localized in the epiphyses of young patients. Damage to the growth plate can lead to limb discrepancy and/or deformities [9]. Conversely, damage of the joint cartilage and the subchondral bone could result in collapse of the joint surface. In the current report, the reconstruction method involved the use of low-porosity β-TCP which has strong compressive resistance. Once implanted, low-porosity β-TCP remains stable throughout the follow-up period [7].

Among the merits of using bone cement as a filler are that rehabilitation can start early and weight bearing is permissible immediately after surgery. Although bone cement lacks the remodeling ability of normal bone and poses the possible risk of degenerative changes in the joint cartilage when there is a subchondral lesion [6], there are other benefits. Bone cement and the resulting heat from the exothermic polymerization process can serve as an adjuvant [2]. Nevertheless, these adjuvants should be used carefully because of possible damage to articular cartilage [4].

As a filler after curettage, a bone graft or β-TCP can offer remodeling ability. However, the mechanical strength of bone after implantation may be lower than normal until such time as when bone incorporation is adequate. β-TCP is a porous bioactive ceramic and can be used as a bone substitute. After implantation, the strength of β-TCP increases in a linear manner according to bone incorporation. Low-porosity β-TCP has a mechanical strength that is initially stronger than normal cancellous bone [11]. A reconstruction method that uses low-porosity β-TCP blocks has been reported for GCTB in the knee [7]. Rehabilitation after β-TCP block reconstruction can start after bone consolidation in the subchondral space, without waiting for consolidation of the whole lesion [7].

In summary, the proximal chondroblastoma case underwent postresection reconstruction using low-porosity β-TCP, which has strong compressive resistance. The reconstruction method described is applicable for humeral chondroblastomas.
